# Peripheral material perception

**DOI:** 10.1167/jov.24.4.13

**Published:** 2024-04-16

**Authors:** Shaiyan Keshvari, Maarten W. A. Wijntjes

**Affiliations:** 1 Department of Psychology, York University, Toronto, ON, Canada; 2 Perceptual Intelligence Lab, Industrial Design Engineering, Delft University of Technology, Delft, Netherlands

**Keywords:** peripheral vision, material perception, texture, modeling

## Abstract

Humans can rapidly identify materials, such as wood or leather, even within a complex visual scene. Given a single image, one can easily identify the underlying “stuff,” even though a given material can have highly variable appearance; fabric comes in unlimited variations of shape, pattern, color, and smoothness, yet we have little trouble categorizing it as fabric. What visual cues do we use to determine material identity? Prior research suggests that simple “texture” features of an image, such as the power spectrum, capture information about material properties and identity. Few studies, however, have tested richer and biologically motivated models of texture. We compared baseline material classification performance to performance with synthetic textures generated from the Portilla-Simoncelli model and several common image degradations. The textures retain statistical information but are otherwise random. We found that performance with textures and most degradations was well below baseline, suggesting insufficient information to support foveal material perception. Interestingly, modern research suggests that peripheral vision might use a statistical, texture-like representation. In a second set of experiments, we found that peripheral performance is more closely predicted by texture and other image degradations. These findings delineate the nature of peripheral material classification.

## Introduction

**Figure 1.**
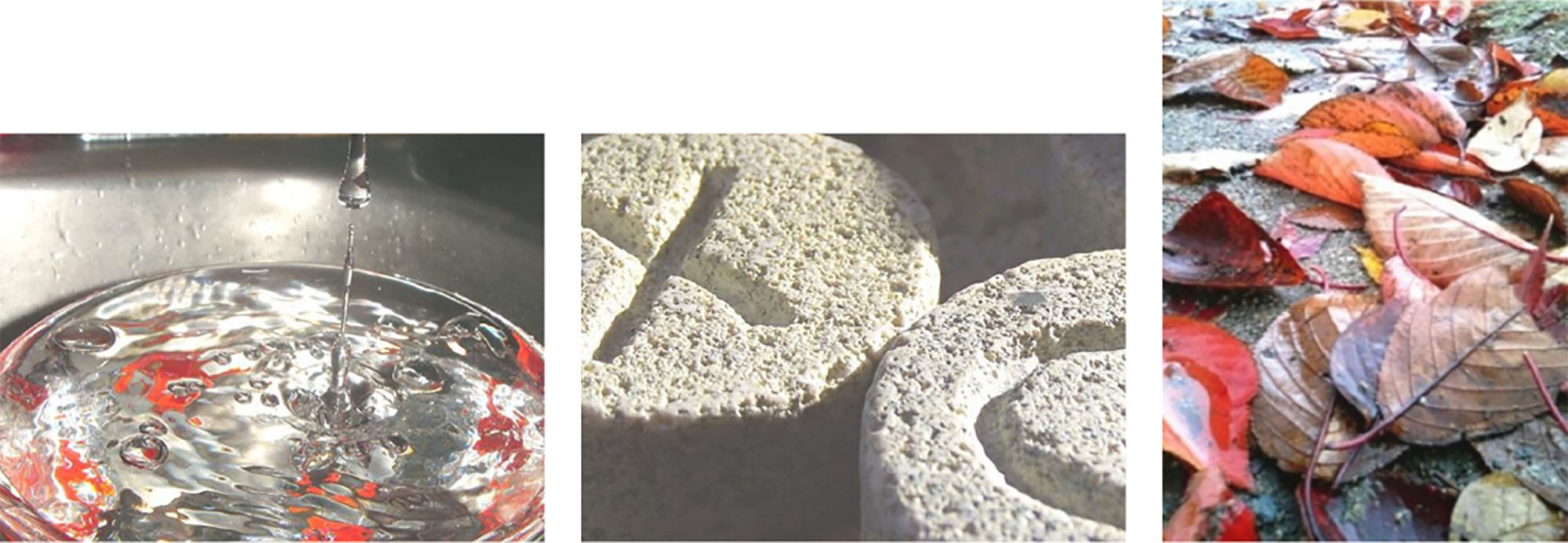
Example material images from the MIT-Flickr Materials Database (Sharan et al., 2014). The images come from the water, stone, and foliage categories, respectively. Notice the large range in viewpoint, illumination, scale, and context.

From a single image, humans can extract detailed information about the properties of a material. Looking at the photograph in Figure 1a, it is immediately obvious that the pertinent material is water. The scene also looks dynamic; droplets and waves suggest how the water got to be there and where it might go next. It is perhaps also apparent that the action is taking place inside a steel sink.

The ability to perceive material qualities allows for rich scene descriptions and for disambiguating between objects of the same category (“the wooden plate”). Furthermore, material perception allows us to safely navigate the world, interact with objects, and infer physical processes. Being able to reliably and quickly tell whether a patch of road is wet or dry is vital for driving, biking, and running, for instance. When grasping soft-serve ice cream, we naturally reach for the solid waffle cone, rather than the creamy filling. Mistaking the material properties of the ice cream could lead to a mess. A photo of squash easily reveals whether it is raw or cooked, solid or pureed, fresh or rotten.

A central goal of research on the perception of materials, or the “stuff” that makes up the world (Adelson, 2001), is to discover what image cues and computations allow humans to determine material properties. Fleming (2014) argues that models of material perception have fallen into two broad categories. First are inverse optics models. In general, these models assume that the visual system simulates or inverts the physical processes by which light reflects off materials and reaches the retina. Combining this internal understanding of light propagation with observations and prior assumptions would allow the visual system to estimate the latent properties of the material. Another class of models posits instead that the visual system relies on statistical regularities in the retinal image, or its “statistical appearance,” to infer material qualities. For example, it has been hypothesized that the skewness of an image's luminance histogram is a cue to the glossiness of the material (Motoyoshi, Nishida, Sharan, & Adelson, 2007). It is clear that this statistic is not the only cue used to determine glossiness (Kim & Anderson, 2010), leaving the door open for further exploration of diagnostic image statistics. One key question is how much or how little statistics are needed. Here, we study to what extent an image-processing and statistics-based model of visual texture can account for material perception.

Visual textures (simply “textures” for short) are loosely defined as images or regions of images with homogenous appearance. A pile of leaves, a striped shirt, and random pixel noise all give rise to texture-like images. It is important to note explicitly the distinction between textures and materials; texture refers to a regular, statistical pattern found in an image. Material, on the other hand, is a richer concept, which may depend on non-texture cues. To illustrate: whether a tabletop is made of wood or plastic may not be obvious from the texture seen in a photograph. Our ability to categorize its material may depend on specular highlights or overall shape. Textures have been studied for understanding perceptual organization (Rosenholtz, 2014), shape perception (Forsyth, 1997), and most relevantly for this study, material perception (Balas, 2017; Balas & Greene, 2023; Balas, 2015; Fleming, 2014; Sharan, Liu, Rosenholtz, & Adelson, 2013; Sharan, Rosenholtz, & Adelson, 2014). There is a long history of modeling human texture processing, and models fall into two general classes: object, or “texton” based models, and image-processing based models. The former posits that the basic elements of texture are individual features, such as angles, endstops, intersections, and more, and that the more different two textures are in their textons, the more perceptually different they will be. The latter family of image-processing based models has gained ground recently for applications in computer graphics (Efros & Freeman, 2001; Gatys Ecker, & Bethge, 2015; Portilla & Simoncelli, 2000) and computer vision (see Rosenholtz, 2014 for a review) and promises to be a more general approach because it operates directly on the image itself, rather than using hand-labeled features. It is pertinent to note that there is extensive prior work on the usefulness of texture models for material classification by humans and computers (Gibson, 1986). The present study considers a specific texture model, known as Portilla-Simoncelli (Portilla & Simoncelli, 2000), which represents a texture using a high-dimensional set of image statistics that are thought to be relevant for early human vision (Freeman, Ziemba, Heeger, Simoncelli, & Movshon, 2013; Freeman & Simoncelli, 2011; Ziemba, Freeman, Movshon, & Simoncelli, 2016) and peripheral vision (Balas, Nakano, & Rosenholtz, 2009; Ehinger & Rosenholtz, 2016; Keshvari & Rosenholtz, 2016; Rosenholtz, 2011; Rosenholtz, Huang, & Ehinger, 2012; Rosenholtz, Huang, Raj, Balas, & Ilie, 2012). Thus P-S statistics seem to be an interesting candidate to represent an intermediate stage between very low-level image statistics, like skewness, and a high-level account, such as inverse optics, of material perception.

 There are several studies on texture for material perception. Sharan et al. (2014) found that the information encoded by a nonparametric texture model was not sufficient to capture material categorization performance. Specifically, they generated textures from material images using the patch-based synthesis method in Efros & Freeman, 2001. Observers viewed these textures for one second and classified them into one of nine categories. First, the observers were significantly less able to classify these textures than the original material images, and performance with the originals was near ceiling. Second, there were significant differences in categorization performance among the nine different material categories when viewing textures.

On the other hand, in a detailed study on machine classification of materials, Sharan et al. (2013) found that small and large-scale texture-like descriptors were essential for good classification performance. Specifically, they computed *jet* (Koenderink & van Doorn, 1987) and *SIFT* (Lowe, 1999) features, measures of color distribution, material shape (in the form of the curvature of edge maps), in addition to reflectance-based features, on material images and filtered versions of them. They trained a classifier to map the feature set from each image into a material category. They found that SIFT was the single best feature for classification, although other features were quite important when included. This suggests that such features are diagnostic of material category. In a relevant extension, they measured the ability of their algorithm and humans to classify textures generated from material images. That is, they scrambled material images using texture synthesis (Efros & Freeman, 2001) (in a similar fashion to their other study [Sharan et al., 2014]) and presented those to their algorithm and human observers. They found that these texture images were not only harder to classify for both the algorithm and humans but nearly equally difficult for both algorithm and human. This suggests potential similarities in encoding.

Although humans are clearly able to extract a much richer representation of a material than simply its identity, we choose to focus on classification ability. Classification presumably relies on many material properties and is less dependent on biases or preferences of observers than subjective judgments. Classification is also arguably an important task for vision and can inform the estimation of other properties. For example, knowing that an object is made of glass reveals something about its material qualities like gloss, roughness, and color (Fleming, Wiebel, & Gegenfurtner, 2013). On the other hand, it is also true that knowing an object's material properties is informative about its identity. The importance of classification is reflected by a growing body of work on material classification by humans, which we summarize here.

Sharan (2009) and Sharan et al. (2014) found that humans can classify images of materials reliably and quickly, despite large variations in color, scale, and context. Critically, they found that this ability does not depend on a single cue, such as color, shape, or small-scale texture; it is rather a more basic and holistic ability. Using the same MIT-Flickr material image database Sharan et al. (2014) and Fleming et al. (2013) found that k-means clustering on averaged subjective ratings of intermediate material properties (like glossiness or transparency) of the images could correctly classify materials with 90% accuracy. This suggests that subjective observations and objective categories are closely connected and that what we learn from categorization experiments will generalize to material perception in general.

Our study's purpose is threefold. First, we test whether a state-of-the-art model of texture, P-S texture statistics, can fully capture what aspects of an image are cues to its material category. This can be understood as an important extension of the texture work done by Sharan et al. (2013) and a rigorous assessment of the extent to which texture supports material perception. It is important to note that although Sharan et al. (2013) did test an image quilting-based texture model (Efros & Freeman, 2001), that model had several shortcomings. First, the output varied greatly with a hand-tuned patch size parameter, which strongly affected categorization ability. Second, because the quilting model synthesizes textures either by tiling random patches or by choosing neighboring patches to be similar, larger structures that span multiple patches are unlikely to be reproduced, leading to a very fragmented image with “hallucinated” edges not present in the original material. The P-S texture model, which is not patch based and measures multi-scale and multi-orientation features, is likely to generate different and possibly more accurate predictions.

Second, we test the ability of observers to classify material images shown peripherally. Since P-S texture statistics are the backbone of a couple of powerful models of peripheral vision (Balas et al., 2009; Freeman & Simoncelli, 2011), it is interesting to compare texture performance to peripheral performance in material perception. Notably, Balas, Conlin, and Shipman (2016) compared material categorization performance between a color version of P-S and peripheral vision. One can consider our study as an answer to the limitations and potential extensions they raise about their own study. Our study differs in methods, analysis, and findings. A full comparison of our studies is complex and not the main goal of this article; therefore we discuss the differences extensively in the Appendix. Moreover, we believe that understanding peripheral material recognition contributes to a general understanding of material perception. An important reason to recognize materials is to facilitate interaction. When we touch or grab something, we have an estimate of the target's properties (Hayhoe, 2017). Manual interaction is not always immediately preceded by foveal scrutiny (L. E. Brown, Halpert, Goodale, Halpert, & Goodale, 2005; Mennie, Hayhoe, & Sullivan, 2007) and likely often based on peripheral information. This provides extra motivation to understand what information is available to the visual system at these moments preceding interactions.

Finally, we compare human performance to several other common image degradations. Namely, we subject the material images to blur, high-pass filtering, phase-scrambling, and analysis/synthesis with the Texture Tiling Model (Balas et al., 2009). The texture tiling model (TTM) is a biologically plausible model of peripheral vision that posits “pooling regions,” which tile the visual field, partially overlap in spatial extent, grow with visual eccentricity, and encode P-S statistics. These image manipulations augment our goal of determining how texture captures material appearance.

## Baseline and texture methods

### Material images

We used images from the MIT-Flickr Materials Database (Sharan et al., 2014). The MIT-Flickr database has several advantages for our purposes over other material image databases. Prior work has shown that people are quite good at categorizing the images in the database (Sharan et al., 2014), despite the large variability in color, pose, scale, semantic content, and illumination within and between material categories. Furthermore, since the images are highly variable, whatever image cues the observers use to do the task are more likely to generalize to real world perception. Prior material image datasets are usually too restricted in viewpoint, illumination, color, content, and more, to be useful for probing general material perception ability. The materials in CUReT (Dana, van Ginneken, Nayar, & Koenderink, 1999), for example, are laid out flat, illuminated identically, and photographed from a single viewpoint and distance. Using such a restricted set might erroneously lead to the conclusion that texture is sufficient for material classification. For example, a texture model might not be able to capture the essential qualities of glass if different lighting angles are used for different exemplars.

**Figure 2.**
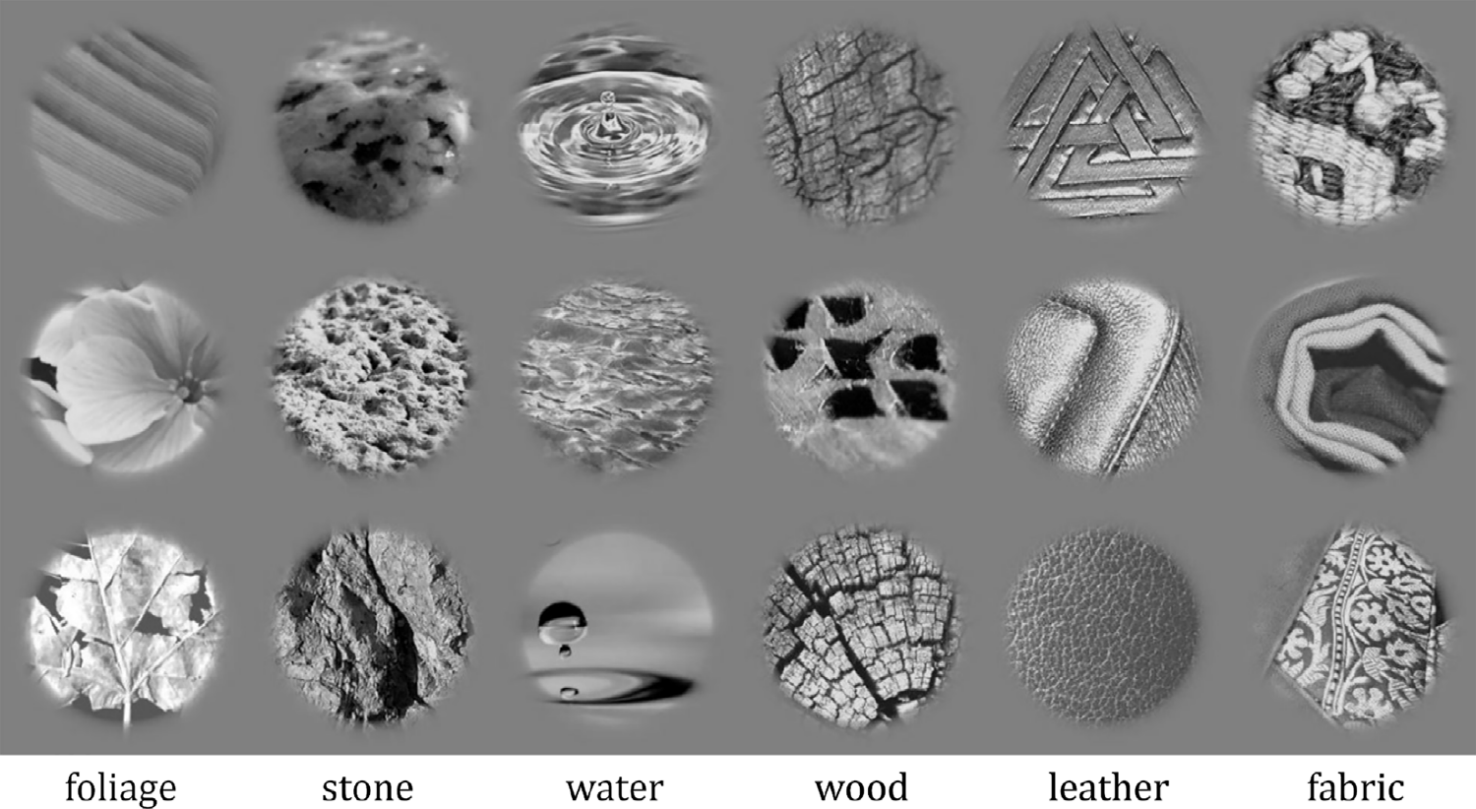
Example stimuli used in the baseline experiment. The label at the bottom indicates the category from which the materials above it were drawn.

The MIT-Flickr database contains a total of 900 images, with nine material categories and 100 images in each category. We only used six of the categories (stone, water, wood, fabric, foliage, and leather; leaving out metal, plastic, and glass) to facilitate an easier six- rather than nine-alternative forced choice task. For each category, the database contains 50 “close-up” and 50 “object-level” images. The object-level images usually contain more background (of a different material than the relevant one), which is problematic for texture synthesis techniques that assume a single texture. Therefore we left those out of this study, resulting in 300 total material images. When generating synthetic textures, we chose to convert all images to grayscale and windowed them, for reasons described in Texture Synthesis. This resulted in material images of 192 × 192 pixels; this same size was used in all experiments. We similarly gray-scaled and windowed material images for the baseline experiment. This helped ensure that differences in performance between baseline and texture conditions are driven by the texture representation. See Figure 2 for some example stimuli.

### Texture synthesis

Textures are generated using the basic P-S synthesis algorithm (Portilla & Simoncelli, 2000). We first converted the images to grayscale by converting to CIELab space and keeping only the luminance channel. This was done for three reasons: first, observers are nearly as good at foveal material classification in grayscale as with color (Sharan et al., 2014), so it is not essential for material perception. Second, there is not a color version of the texture model that is widely accepted and tested with respect to human vision (although a color version exists at http://www.cns.nyu.edu/∼lcv/texture/). The grayscale P-S model has been tested extensively with a variety of stimuli and tasks (Balas et al., 2009; Rosenholtz, 2017). Third, we wish to avoid a scenario in which observers rely only on color and thus the results do not reflect the influence of texture. Then, for each material image, we compute P-S statistics (with default parameters) and use the P-S synthesis algorithm along with a random noise seed to generate a synthetic version of the same size. We run the algorithm for many iterations (150) to ensure convergence. This procedure results in 300 synthetic textures, one for each original material image.

**Figure 3.**
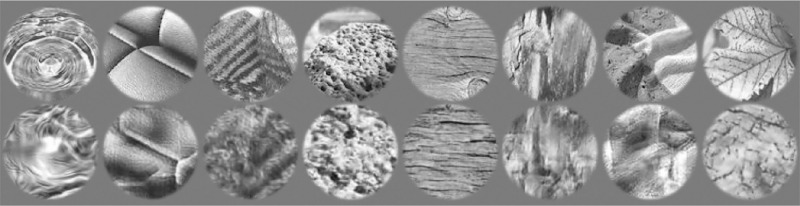
Example materials along with their synthetic texture versions. Each image in the first row column is the original, and the corresponding image in the second row is a sample texture.

The algorithm assumes that the image wraps around top-to-bottom and side-to-side (i.e., it assumes the original and synthesized images lie on a torus). This means that the edges of the image are nonintuitively structured, and it is cleaner to leave them out by windowing (mentioned in the previous subsection). Note that the windowing is done after synthesis (textures are computed over the whole, un-windowed material image; the synthesis produces a full-sized image that is then windowed). We used a circular window of 2° visual angle in radius, with a smooth Gaussian fall off of standard deviation of 0.5 deg (see Figure 2). Figure 3 illustrates some materials and their texture counterparts.

### Observers

Sixteen observers participated in the experiments. Five observers did the baseline experiment, and 11 did the texture task (these latter observers also did the peripheral task in a separate block as discussed in the Peripheral section). All observers were naïve as to the purpose of the experiment, and all had normal or corrected-to-normal vision. Observers in the baseline experiment were paid $10 for about 30 minutes of experiment; the rest were paid $15 for about an hour of experiment.

### Apparatus

Stimuli were presented using Psychtoolbox 3 (Kleiner et al., 2007) and MATLAB on a CRT monitor with a mid-gray background. Observers used a chinrest in all conditions. Observers responded by using a mouse to click on one of six circles, each labeled with one of the categories.

### Procedure

For all experiments, observers had unlimited viewing and response time. Observers were first oriented to the task by the experimenter and shown example stimuli along with category labels. The experimenter also informed them that all categories occurred with equal frequency, and to do the best possible without spending too much time on a particular trial. Observers received feedback on the first 25 trials. Each observer saw all 300 unique stimuli, in random order.

**Figure 4.**
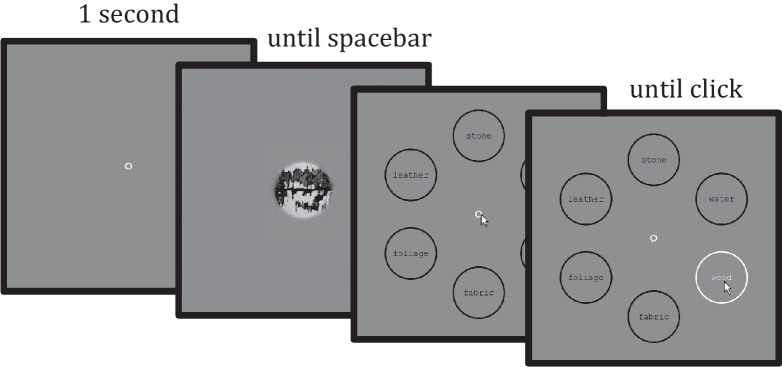
Procedure for a single trial in the baseline experiment. If the trial contains feedback, it is displayed immediately after clicking. The procedure for the texture experiment is identical, except displaying a texture image instead of a material.

For each trial, the procedure is as follows: the stimulus (material image) appears in the center of the screen after a one- second central fixation (fixation circle remains on throughout each trial). The observer then has unlimited time to push the spacebar to end the presentation. Upon pressing the spacebar, the stimulus is removed, and the decision screen appears. On the decision screen, the name of each material category is shown in a circle of radius 2°, at 8° eccentricity (evenly spaced in a notional circle centered at the screen center). The observer then moves the mouse to the desired choice and clicks to make a response, and the decision screen is removed. The first 25 trials have visual feedback as to correctness of the response (a change in the color of the central fixation for 0.5 seconds). During the rest of each experiment, observers receive no feedback, with the fixation remaining white for 0.5 seconds after the response. The next trial begins after the feedback/white fixation. The observer receives an untimed break every 75 trials. A schematic of the procedure is illustrated in Figure 4.

### Baseline

For the baseline task, observers viewed all 300 windowed material images presented foveally and did the classification task as described in the Procedure section. It is important to get a baseline measure of performance for several reasons. If we are to examine texture as a cue for material category, we need to know how well observers can tell category with all cues present (i.e., the original materials). To our knowledge, there has not been a study of untimed, grayscale material recognition with the MIT-Flickr database using the subset of images we choose here. Importantly, it is not obvious that observers will be perfect at this task. The images come from a wide range of three-dimensional shapes, object identities, surface reflectances, physical scales, and illuminations, even within a category. Our later experiments compare performance in this baseline condition to performance under degraded viewing conditions. If observers are less able to categorize materials with the textures than the baseline, this would imply that texture is not a sufficient cue for category. In other words, this finding would imply that the information lost by converting a baseline material to a P-S texture (e.g., shape or large-scale layout information) is necessary for robust material classification. If, on the other hand, texture classification performance is indistinguishable from baseline performance, we cannot draw definitive conclusions about the necessity of texture for material classification.

### Texture

For the texture task, observers foveally viewed windowed synthetic textures generated using the procedure described in the Texture Synthesis section. Specifically, the observers did the same classification task as described in the Procedure section, except viewing textures. The experimenter gave a colloquial explanation of how the textures are generated from the original materials, including that parts of the image might be translated, swapped, and mixed with respect to the original material image. Importantly, they were instructed to respond to what material category the texture was generated from, rather than what material the texture itself might look like. This distinction encourages the observer to do as well as possible with the available information and consider their intuitions about the texture-generation process. Also, this discourages the observers from interpreting artifacts caused by the synthesis process as informative cues. The goal is to use human observers to measure what classification is possible, given only the texture statistics (Balas et al., 2009). For this purpose, we want them to make full use of the available information, which includes bringing to bear understanding of the effects of texture synthesis on images of materials.

## Baseline and texture results

We analyzed the results in several ways. First, we discuss the results from each experiment separately, and then compare them. Unless otherwise noted, statistical significance values are computed using a Bonferroni-corrected two-sided random-permutation test. Such nonparametric permutation tests are more appropriate than traditional hypothesis tests, for example *t*- or *F*-tests, because categorization tasks violate the necessary normality assumptions (Still & White, 1981).

### Baseline

**Figure 5.**
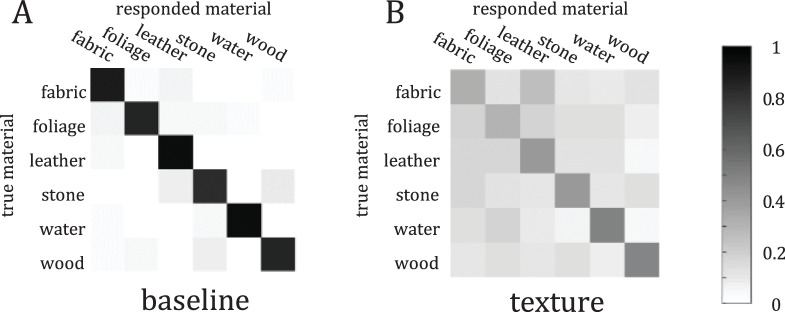
Confusion matrices for the baseline (A) and texture (B) experiments. The row indicates the true material, and the column indicates the response. Darker shades indicate a higher prevalence of the response. The color bar on the right provides a reference for exact values. Notice that most of the mass is along the diagonal in the baseline case, indicating high performance. In the texture case, the diagonal is weaker, and errors are widely distributed.

Five observers completed the baseline experiment, each doing 300 trials. The average performance (proportion correct) over all six categories and all subjects was 0.882 (chance is 1/6 ≈ 0.167). Each observer performed well above chance in each category (*p* < 2 × 10^−5^). This is in line with previous work, where Sharan et al. (2014) found that observers’ performance was 0.866 (chance is 1/9 ≈ 0.11) for nine-way grayscale material classification. A confusion matrix of responses is shown in Figure 5A.

**Figure 6.**
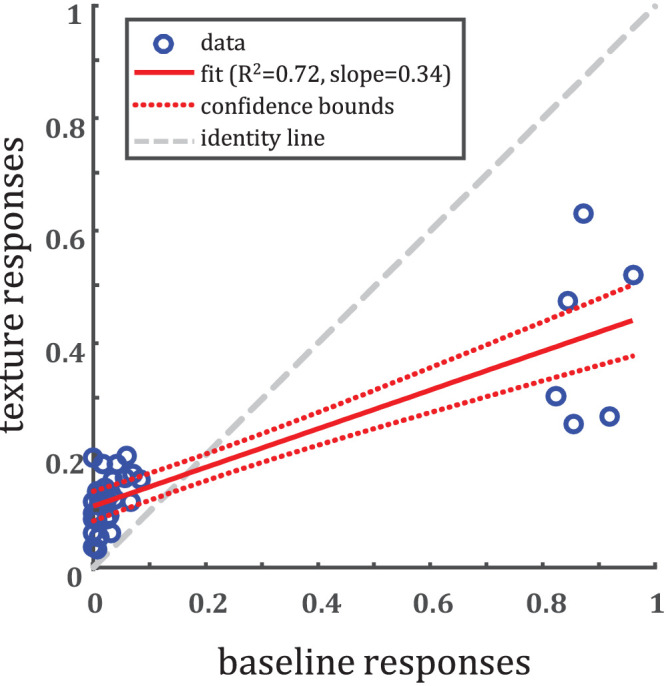
Comparison of elements in the texture confusion matrix (Figure 5A) to elements in the baseline confusion matrix (Figure 5B). The cluster of points on the right are the diagonal elements, the proportions of correct responses. Notice that although baseline is not well predicted by texture, texture performance is above chance (1/6).

### Texture

Eleven observers completed the texture experiment, each doing 300 trials. The same subjects did the peripheral experiment in a different block. Average performance over subjects and categories was 0.40. Each subject performed above chance (*p* < 2 × 10^−5^) averaging over all categories. Each subject was also above chance within each category (using a *p* < 0.05 criterion), with a few exceptions: two subjects were not above chance at classifying fabric, three observers were not above chance with foliage, and one observer was not above chance with leather. The confusion matrix is shown in Figure 5B.

### Comparison

We directly compare the elements in the confusion matrix from one experiment to the other (see Figure 6). Note that since these “data points” do not obey the assumptions made by regression (independent errors, homoscedasticity, and weak exogeneity), the *R*^2^ value is not strictly appropriate. Nevertheless, the strong correlation intuitively would indicate not only that the “correct” responses are similar between conditions, but so are the confusions (off-diagonal elements).

Fitting a simple linear regression model to all the elements, we find significant correlation (adjusted *R*^2^ = 0.72). The fitted slope (slope = 0.34), however, is not close to the identity line (slope = 1, intercept = 0), as we would expect if the experimental conditions were comparable. For this reason, we argue that baseline performance is not well predicted by performance using the texture images.

### Interim discussion

We can draw several conclusions from the baseline and texture experiments. First, humans are excellent at categorizing grayscale images of materials. Performance is near ceiling for each subject in each category. Second, performance in the texture task is much lower than ceiling. Although all subjects are performing above chance, for certain categories, there are subjects that do not perform above chance. This implies that texture is a cue to material category, although it is not sufficient to explain baseline categorization ability.

Comparing baseline to texture performance, we see that texture statistics are not sufficient to convey material category. It is difficult to assess whether the pattern of classification errors is the same in the two conditions because there are few classification errors in the baseline condition. In spite of the fact that texture clearly provides one cue to material perception (Rosenholtz, 2014) and that the texture model we used is a top-performing model of texture perception, performance with texture-only material images was well below that of the baseline condition. This suggests that other cues must play a large role in classification.

## Peripheral vision

Humans can identify materials peripherally, for example, when noticing wet leaves on a road while driving and looking ahead. This plays into rapid scene categorization where observers quickly classify a natural scene. Research on rapid scene categorization argues that this ability depends on low-level image cues (Oliva & Torralba, 2001) rather than by identification of objects in the scene. This finding is relevant to the present study for two reasons: First, because of the rapid presentation, most of a scene can only be seen peripherally. Second, in natural scenes, most of the image consists of regions of various materials rather than individual objects. A waterfall scene, for example, might have water running along the middle with foliage and stone on the sides; there may not even be easily individuated plants or rocks.

Furthermore, there is an established body of work modeling peripheral vision as forced texture perception. This line of research suggests that a statistical representation that pools information over large regions of the visual field, namely visual texture, captures the information available to peripheral vision. Visual texture has made successful predictions for many peripheral vision phenomena (Rosenholtz, 2011), such as crowding (Balas et al., 2009; Keshvari & Rosenholtz, 2016), visual search (Chang & Rosenholtz, 2016; Rosenholtz, Huang, & Ehinger, 2012; Rosenholtz et al., 2012; Zhang, Huang, Yigit-Elliott, & Rosenholtz, 2015) and scene perception (Ehinger & Rosenholtz, 2016). As mentioned previously, one study has even made an examination of peripheral material perception with a subset of material categories (Balas et al., 2016). For these reasons, we explicitly test peripheral material classification, and compare it to results from both baseline and texture material classification.

### Peripheral methods

In the peripheral task, observers viewed windowed grayscale material images (not textures) at 10 deg center-to-center eccentricity. The images were randomly shown to the left or right of fixation. We used the Eyelink 2000 (SRI Labs) for eyetracking, along with the standard built-in calibration procedure. The image was only on while the observer was within 2° of the central fixation; if the gaze fell outside of the central 2°, the image was removed until the gaze returned. Eyetracking was not used during the decision stage. Thus the peripheral condition was identical to the baseline condition except that the material images could only be viewed peripherally.

### Peripheral results

**Figure 7.**
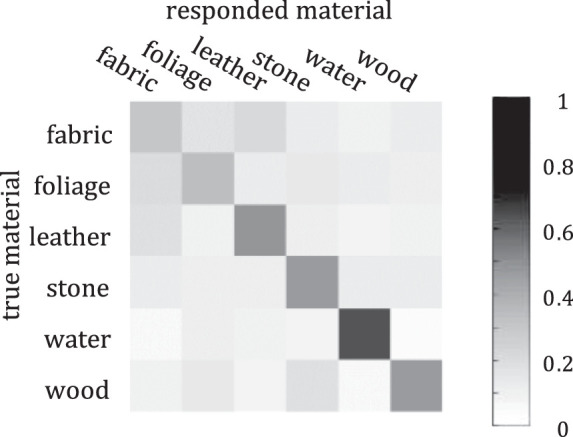
Confusion matrix for peripheral viewing experiment. Similar to the confusion matrix for texture, most of the responses fall along the diagonal (indicating correct classifications), and there is a large spread in the errors (off diagonal elements).

Eleven observers completed the peripheral experiment, each doing 300 trials, in the same session but different block as the texture experiment. Block order was counterbalanced among subjects. Average performance over subjects and trials was 0.44. As in the texture experiment, all subjects performed well above chance when averaging over all categories (*p* < 2 × 10^−^^5^). For each subject and category, performance was above chance (*p* < 0.05) except for one subject for fabric, one for foliage, and one for leather. As before, the confusion matrix of responses is shown in Figure 7.

#### Comparisons

##### Confusion matrix comparison

**Figure 8.**
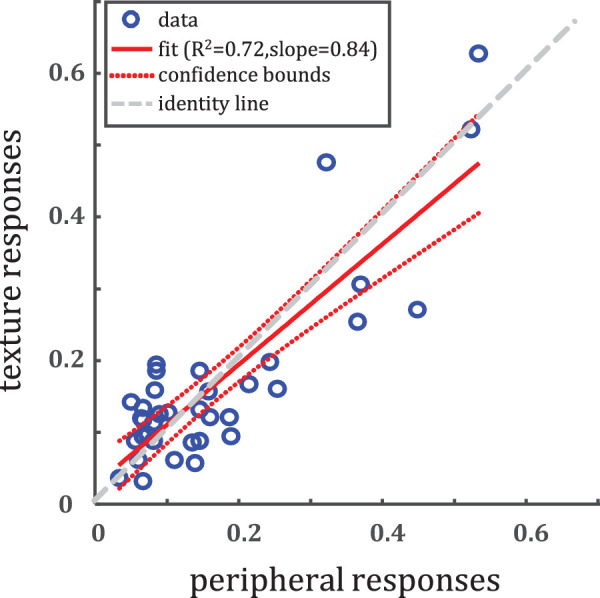
Comparison of elements in the texture confusion matrix (Figure 5B) to elements in the peripheral confusion matrix (Figure 7). Notice that not only correct classifications (the values above about 0.3 in both dimensions) fall close to the diagonal; off diagonal elements (confusions) do as well.

Comparing the confusion matrices between the baseline and peripheral, we find that baseline performance is also not well predicted by peripheral; this is unsurprising given the large discrepancy between overall performance. Comparing the elements in the matrices between texture and peripheral experiments, however, we find strong correlation (*R*^2^ = 0.72, slope = 0.84; Figure 8).

## Online experiments

These findings suggest an important role for texture in perception of materials in the periphery. To get a more complete picture of the roles played by other possible image properties, we conducted five online experiments testing alternative cues for material perception.

### Online methods

All experiments used grayscale material images, altered in the ways described below, and windowed with the same parameters as the in-lab foveal experiment. The five image manipulations are as follows:

**Blur:** Material images are convolved with a gaussian kernel of standard deviation of 8 pixels (approximately 4.17% of the image size).
**High-pass:** Each image is blurred with a gaussian kernel of standard deviation of four pixels (approximately 2.08% of the image size), then subtracted from its original.
**Phase-scrambled:** Images are transformed with Matlab's fft2, and the phase image is shuffled randomly before recombining with the unchanged magnitude image using ifft2.
**Texture:** The images are identical to the synthetic texture images used in the lab experiment.
**TTM:** Each image is analyzed and synthesized using the Texture Tiling Model (Balas et al., 2009) using default synthesis parameters and the same eccentricity as in Peripheral methods.

For each image manipulation, 40 unique observers were recruited from Amazon Mechanical Turk. Observers gave informed consent and were compensated for finishing the experiment. There was no time limit enforced. Each observer classified all 300 images, as in the lab experiment, split equally among the six ground truth categories and ordered randomly. Observers were oriented to the experiment with examples of the original and manipulated images. They had unlimited time with each stimulus. To ensure that participants were engaged with the task, we randomly interspersed six catch trials; instead of classifying an image for these trials, observers responded to a question about each of the categories (i.e., “Which category includes leaves and plant material?”). Any observer who responded incorrectly for more than one of the catch trials was excluded from analysis. After removing these observers, we were left with the following: 25 observers for blur, 31 observers for high-pass, 25 observers for phase-scrambled, 24 observers for texture, and 26 observers for TTM.

### Online results

#### Average performance

Average performance in the tasks was different for each experiment, ranging from 0.35 with TTM images to 0.62 with high-pass images. See Table 1 for performance in each online experiment.

**Table 1.** Performance in online experiments. See text for numbers of subjects in each experiment.

Experiment type	Blur	Texture	Phase-scrambled	High-pass	TTM
Mean performance	0.43	0.36	0.45	0.62	0.35
Standard error	0.018	0.023	0.023	0.030	0.012

#### Comparisons

When comparing results between observers in different experimental conditions, we use the modified Cohen's Kappa developed by (Geirhos, Meding, & Wichmann, 2020). Briefly, the measure κ is defined as the number of times two classifiers make consistent decisions (incorrect or correct) with the same stimulus, normalized by the number of expected randomly consistent decisions simply due to overall performance. This allows us to measure similarity between classifiers in a more granular way. It is limited, however, because it does not consider exactly which confusions are made; only that the response is correct or incorrect.

For each stimulus condition, we measure within-condition $\bar{\kappa }$ by averaging individual κ values computed between all possible pairs of observers. Similarly, we measure between-condition $\bar{\kappa }$ by averaging all possible κ that can be computed between observers in the two different conditions. This requires comparing performance on the same material image between observers and experiment types. Error bars correspond to SEM computed over all possible comparisons.

Comparisons between observers in these image-degradation experiments and observers in the lab experiments reveal several similarities and differences. For example, we find that classification in online experiments with blur, texture, and TTM-degraded images is significantly more like classification in the periphery than in the fovea. Furthermore, performance with high-pass materials is significantly more like foveal material perception than peripheral. Phase-scrambling and texture in-lab trend toward a matching peripheral classification more closely than foveal but are not significant after correcting for multiple comparisons. It is important to reiterate, however, that this analysis only considers how often raters were both correct or incorrect on matched trials, normalized by agreement because of chance; it does not consider the actual responses made.

## Discussion

Materials are ubiquitous in natural visual experience, and humans are remarkably good at identifying materials. This ability is robust to large changes in viewpoint, illumination, scale, color, and subclass (Sharan et al., 2014). We found that texture statistics support this ability to some extent but are not sufficient to explain foveal material classification. While some examples of materials are well-captured by this representation (for example, see the two top right images of wood and their textures in Figure 3), most material examples contain information that is not retained by texture statistics. This failure of texture statistics to explain foveal perception is in line with a previous study, which found that a nonparametric model of texture (Sharan et al., 2013; Sharan et al., 2014) was a poor descriptor of material category to foveally-observing humans and algorithms.

Some materials are better represented as a texture than others. We suspect that this derives from inherently unique statistical properties of the materials. For example, water has sharp caustics, waves, and distortions that are not found as often in the other categories. Furthermore, some materials may have a more diverse appearance. Foliage sometimes consisted of small, texture-like repetitive structures like overlapping leaves or bark, while other times it was a prominent single object or shape, such as a single leaf or flower. In the latter cases, a texture model seemed to perform poorly; it tosses out large-scale shape information, which would otherwise help with categorization. It is interesting to view these results in light of the findings of Sharan et al. (2013) with respect to non-texture features. They find that features measuring variation across and along only edges, including both curvature and so called “edge-slice” and “edge-ribbon” features, are important for machine material classification. These features, which are designed to glean shape and reflectance information, might be a piece of the information missing in P-S. Interestingly, they found these non-texture features be vital for their computer vision system. Along these lines, a more recent instantiation of TTM measures and preserves end-stopping statistics (Brown et al., 2022) defined as squared difference between adjacent, orthogonal edges seems to improve its power in simulating peripheral vision.

**Figure 9.**
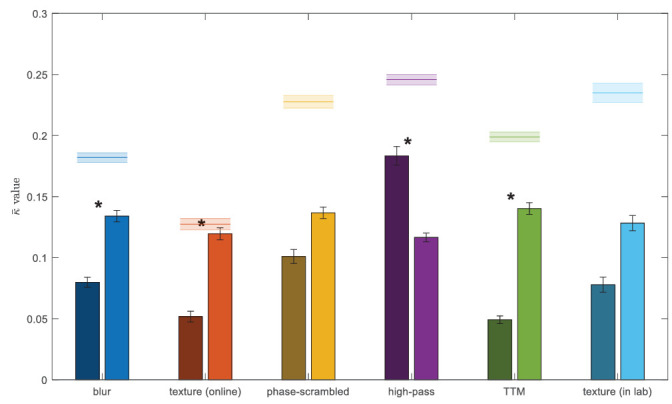
Average inter-rater similarity scores using modified Cohen's kappa (Geirhos et al., 2020). Dark bars in each pair are computed between the listed stimulus condition and foveal observers, whereas the lighter bars are computed between the stimulus condition and peripheral observers. The horizontal line above each pair of bars represents the average self-similarity between observers in the stimulus condition; this can be considered as an upper bound on inter-rater similarity. Asterisks represent significant differences at *p* < 0.05 in a one-way ANOVA with Dunn-Sidak correction for multiple comparisons. Error bars and shaded regions represent standard error of the mean.

One main finding in this study is the similarity in performance between degraded images and peripheral viewing. Namely, we find that the blur, texture (online), and TTM images are classified more like peripheral images, even accounting for similar performance, than other image degradations like high-pass filtering or phase scrambling (Figure 9). This suggests that peripheral viewing of materials decreases performance in a specific way, rather than causing a general increase in difficulty; the correlation between peripheral and texture responses is not due to only stimulus-inherent difficulty. While blurring does match peripheral viewing closer than foveal viewing, note that to get sufficient loss in performance, the level of blur used in this experiment had to be approximately 19 times larger than what would be required to mimic peripheral acuity loss at 10 degrees eccentricity. Specifically, an acuity-matched gaussian blur kernel would have *σ* ≈ 0.42 pixels at 10 degrees eccentricity (Rodieck, 1998), whereas the blur we use has *σ* = 8 pixels. For this reason and blur's inability to model other peripheral phenomena, we do not consider this as evidence for blur as a complete model of peripheral material perception. The P&S texture model and TTM are viable models for peripheral vision and have been shown to predict the peripheral phenomena in many other studies (see Rosenholtz, Yu, & Keshvari, 2019 for a review). It is possible that some weighted linear or nonlinear combination of the models we tested would best predict peripheral material perception; this is a potential avenue for future research. Interestingly, we find a discrepancy in texture's ability to match periphery between in-lab and online studies. We believe this is due to the larger number of observers in the online study, although this merits further study. Finally, as part of the synthesis process, TTM blurs the stimulus a small amount to simulate acuity losses in peripheral vision. This amount of blur may serve to bring its predictions more in line with the periphery. Note that Balas et al. (2016) do not find a close correspondence between textures and peripheral vision of materials; we compare their study to the current one extensively in the Appendix.

It is worth mentioning what might change if color were to be included in this study. It is likely true that our choice of material categories would allow observers to perform above chance with color alone; the six classes we used all have different prior color distributions. Furthermore, the loss of information available in the periphery might actually make color more important in the periphery, because spatial cues become more ambiguous. In fact, given the loss of spatial order, it is possible that randomly using some colors from the target or showing a separate representation of the color distribution peripherally would be as good as using the “correct” color texture model. Although certainly a worthy topic of study, making a definitive statement about the role of color would require specifying a model and conducting one or more further experiments, which falls outside the scope of the current study.

**Figure 10.**
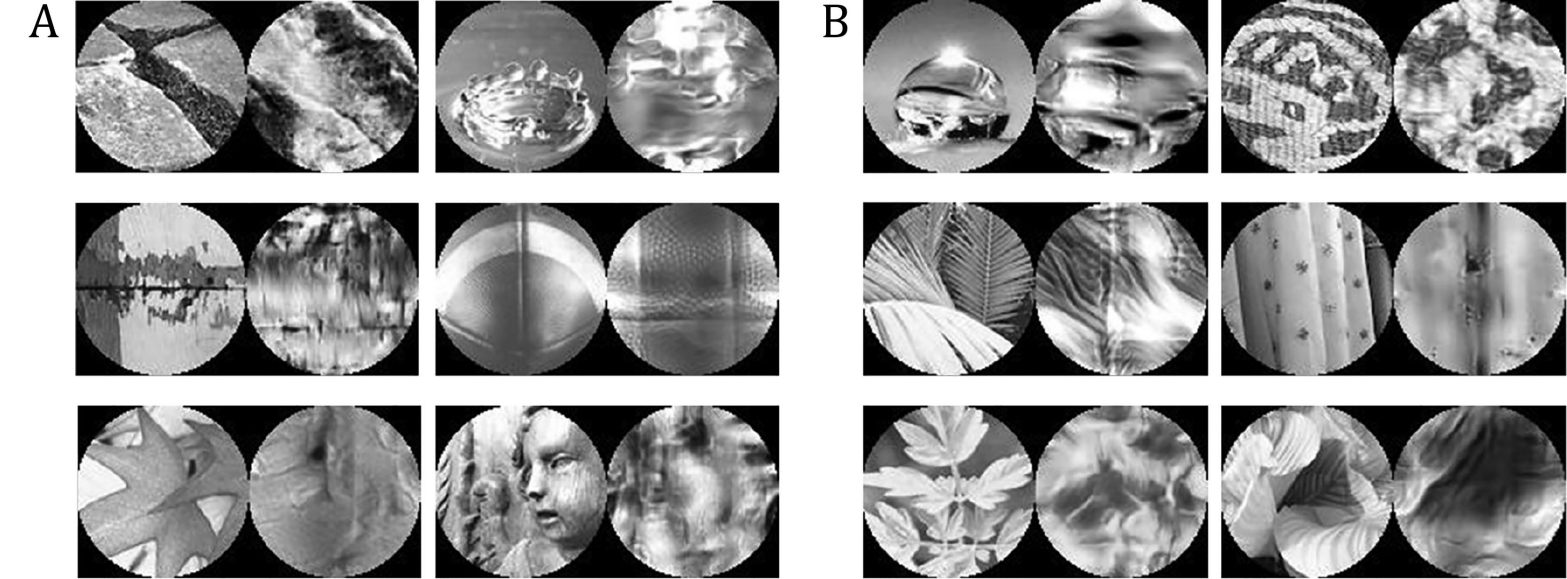
Example materials stimuli with corresponding textures. (A) Materials for which more observers are correct with textures than in the periphery (upper-left region of Figure 8). (B) Materials for which more observers are correct in the periphery than with textures (bottom-right region of Figure 8). The differences between these materials is useful for spotting the shortcomings of texture as a model of peripheral vision.

By showing each material image both peripherally and as a texture to each subject, we are able to visualize per stimulus where the statistical model and peripheral vision differ. We can glean some intuitions by considering stimulus-texture pairs in cases where more subjects were correct with textures than peripherally, and vice versa. In the case where texture performance is better than peripheral performance (Figure 10), there seem to be shape cues which are not diagnostic (or even misleading) of material category. The statue of a child in the bottom right image is made of wood; but statues can be made of several different materials, including stone. By tossing out large-scale shape information, the texture model may actually represent the material identity better. Similarly, for the football in the middle right, the overall curved shape does not necessarily hint at leather; only by “stripping off” the surface material with texture is the leathery look obvious. We can also consider cases where peripheral vision is better. Looking at Figure 10B, the fabric example in the middle right has long-range structure. Namely, the vertical folds give away the fabric nature of the material; these long-range structures are washed out in the statistical representation. These findings suggest that the texture model lacks some of the long-range correlations that peripheral vision picks up on.

One conclusion that we can draw from this study is that a full model of peripheral vision must represent these shape cues with a higher fidelity. This may be done through explicitly adding shape information to the model, as implemented by (Sharan et al., 2013) for machine classification or for visual search (Alexander, Schmidt, & Zelinsky, 2014). It might also be achieved by using multiple, overlapping regions where texture statistics are computed. Extensive and promising work has been done in this domain using such a model, known as the Texture Tiling Model (Rosenholtz, Huang, & Ehinger, 2012). Future research should address whether the long-range correlations afforded by models like the TTM are sufficient to explain peripheral texture perception.

## Figures and Tables

**Figure 11.**
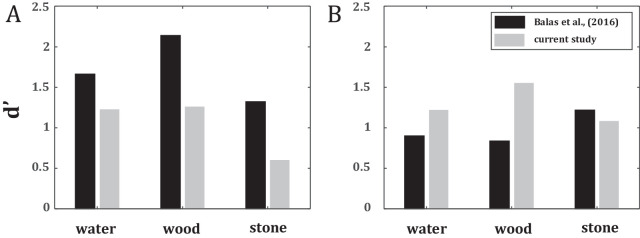
Comparison of material classification sensitivity in Balas et al. (2016) to the current study. The main differences between the experiments are as follows: Balas et al. (2106)’s stimuli subtended 4 degrees visual angle, were colored, uncropped, and displayed only for 250 ms, whereas ours were 2 degrees wide, grayscale, cropped with soft circular edges, and shown for unlimited viewing time. Furthermore, they used an unspecified subset of 25 material images per category from the full FMD database, whereas we used all 50 “non-object” images per category. (A) Sensitivity for water, wood, and stone images viewed in their “far periphery” experiment compared to our “peripheral” experiment (both experiments used 8 degrees eccentricity). (B) Sensitivity for the same categories but comparing their “synthetic” to our “texture” experiment, where stimuli were synthesis using the P-S algorithm and shown foveally.

## Appendix Appendix

We use the appendix to compare in detail our study to Balas et al. (2016). Given that they also conducted experiments on material classification, comparing the periphery to P-S textures, we find it appropriate to go into some detail. Both their and our studies examine the similarity between material and texture perception. They use different methods, however, leading to different findings and conclusions. Importantly, their discussion suggests several shortcomings of their approach, which we believe our study addresses. By addressing these issues, we help complete our understanding of the relationship between texture and material perception.

### Differences in methods

#### Eye movements and stimulus duration

To limit subjects’ use of eye movements, Balas et al. (2016) displayed stimuli only for short times (250 ms), whereas we used eye-tracking to make a gaze-contingent display while allowing unlimited viewing times. This difference in presentation times may lead to differences in performance between our studies; however, to our knowledge, there has not been a systematic study of how stimulus duration affects peripheral perception of materials or other naturalistic stimuli. Many studies vary peripheral stimulus presentation time with artificial or naturalistic stimuli. Some findings preclude significant improvement above 250 ms (Lev & Polat, 2015; Morikawa, 2000; Saarinen, 1988; Thorpe, Gegenfurtner, Fabre-Thorpe, & Bülthoff, 2001; Trouilloud et al., 2020) whereas others do not (Kooi, Toet, Tripathy, & Levi, 1994; Tripathy, Cavanagh, & Bedell, 2014; Tripathy & Cavanagh, 2002). This is a promising avenue for future research on the dynamics of peripheral vision.

#### Stimulus selection

Balas et al. (2016) used 25 unique images per category, and 4 categories, for a total of 100 images per condition; we use 50 unique images per category, and 6 categories, for a total of 300 images per condition. They did not filter out the “object” level stimuli, and do not indicate how they selected specific images from the larger FMD database. The “object” level stimuli are likely to have more global structure, and as suggested by our findings, might be partially responsible for differing results. We observe large variance in performance between individual stimuli, even when leaving out the “object” level stimuli.

#### Stimulus size

The stimulin of Balas et al. (2016) subtended 4° visual angle and were not cropped, whereas ours subtended 2° and were cropped circularly with soft edges. We believe that the sharp edges of their stimuli might increase difficulty when viewing peripherally, potentially by causing visual crowding between the sharp edge and material image.

#### Stimulus color

The original materials of Balas et al. (2016) are shown in full color, and they use the color version of P-S to generate textures; we use grayscale original materials and grayscale P-S. We discuss this difference in detail in the Discussion.

#### Data analysis

Balas et al. (2016) drew conclusions based on comparing correct classifications separately from confusions, between experiments. Our approach does not include statistical tests on the differences in performance between individual categories. Rather, we report the aggregate correlation over all categories and all confusions. We believe that aggregating all of the materials and confusions within an experiment allows for a more comprehensive result.

#### Model parameters

The size of the autocorrelation matrix is a free parameter in the P-S model. Autocorrelation is measured on an original image, and enforced during synthesis. Balas et al. (2016) used a 21- × 21-pixel autocorrelation matrix; we used 9 × 9, which is the default for the model. We could not determine their motivation behind this parameter value choice.

### Differences in findings

To compare the results between our tasks and those of Balas et al. (2016), we computed the d′ (using the dprime.mAFC function in Ken Knoblauch's R package from the confusion matrices, https://cran.r-project.org/web/packages/psyphy/index.html) sensitivity index for each category. This allows us to account for the difference in the number of alternative choices between the tasks (Balas et al. [2016] used four materials while we used six). We compare their “far-periphery” and “synthetic” results to our “peripheral” and “texture” results, respectively, because they are the most similar. Within those tasks, we compare performance among the three materials present in both studies: water, wood, and stone.

Looking at the comparisons in Figure 11, we can see that sensitivity in our task is lower for each common category in peripheral classification, while it is higher for the “synthetic” or “texture” task. As we discussed earlier, there are several competing differences between the experiments, which might push sensitivity one way or the other. For example, we think the fact that their stimuli are twice the size of ours at the same eccentricity (their far-periphery condition), e.g. 4 instead of 2 degrees square, as well as to a lesser extent the inclusion of color, drives their higher sensitivity peripherally. Performance is higher for our “texture” task (than their “synthetic”), despite lacking color and being smaller; we believe that the short display times of Balas et al. (2016) make the already-challenging texture classification task especially difficult for their subjects. To our knowledge, all previous studies using P-S as a model of vision used long display times.

### Differences in conclusions

Balas et al. (2016) draw several conclusions. First, based on their result that four material categories had roughly the same peripheral performance (except wood), they argue that “The lossy transformation imposed by viewing natural images in the periphery therefore does not appear to differentially impact the recognizability of different materials.” We tested a wider range of materials and more unique examples of each material and found there to be large differences in sensitivity between categories. Second, in attempting to explain the difference between the results of the peripheral and synthetic experiments, namely “P-S algorithm does not appear to provide an adequate feature vocabulary for explaining correct material categorizations,” they suggest several explanations. These include (A) “Increased viewing time for the synthetic images, decreased viewing time for the original images, or increased eccentricity of stimulus position for original images could all bring the baseline performance across all categories closer together between these two conditions”; (B) “while the algorithm may not easily reflect how difficult our participants found wood to categorize accurately in the periphery, it may capture the variability across other material categories”; and finally (C) “Presenting observers with grayscale versions of the images used here could lead to a different outcome, again suggesting both an important limitation of the model and an important property of human material perception.” Our study explicitly addresses these issues they raise (note the differences between methods discussed in the Appendix), and indeed finds different results. In a sense, our study is an answer to their “number of intriguing roads to further inquiry.”
